# Supplementary material for: Ontogeny of arterial macrophages defines their functions in homeostasis and inflammation
Source: Nat Commun. 2020 Sep 11;11:4549. doi: 10.1038/s41467-020-18287-x (PMC7486394; doi:10.1038/s41467-020-18287-x)
Supplement: Supplementary file 1 — Supplementary Information [file 41467_2020_18287_MOESM1_ESM.pdf]

Supplementary Information File

Ontogeny of arterial macrophages defines their functions in homeostasis and inflammation

First Author: Tobias Weinberger

Corresponding Author: Christian Schulz

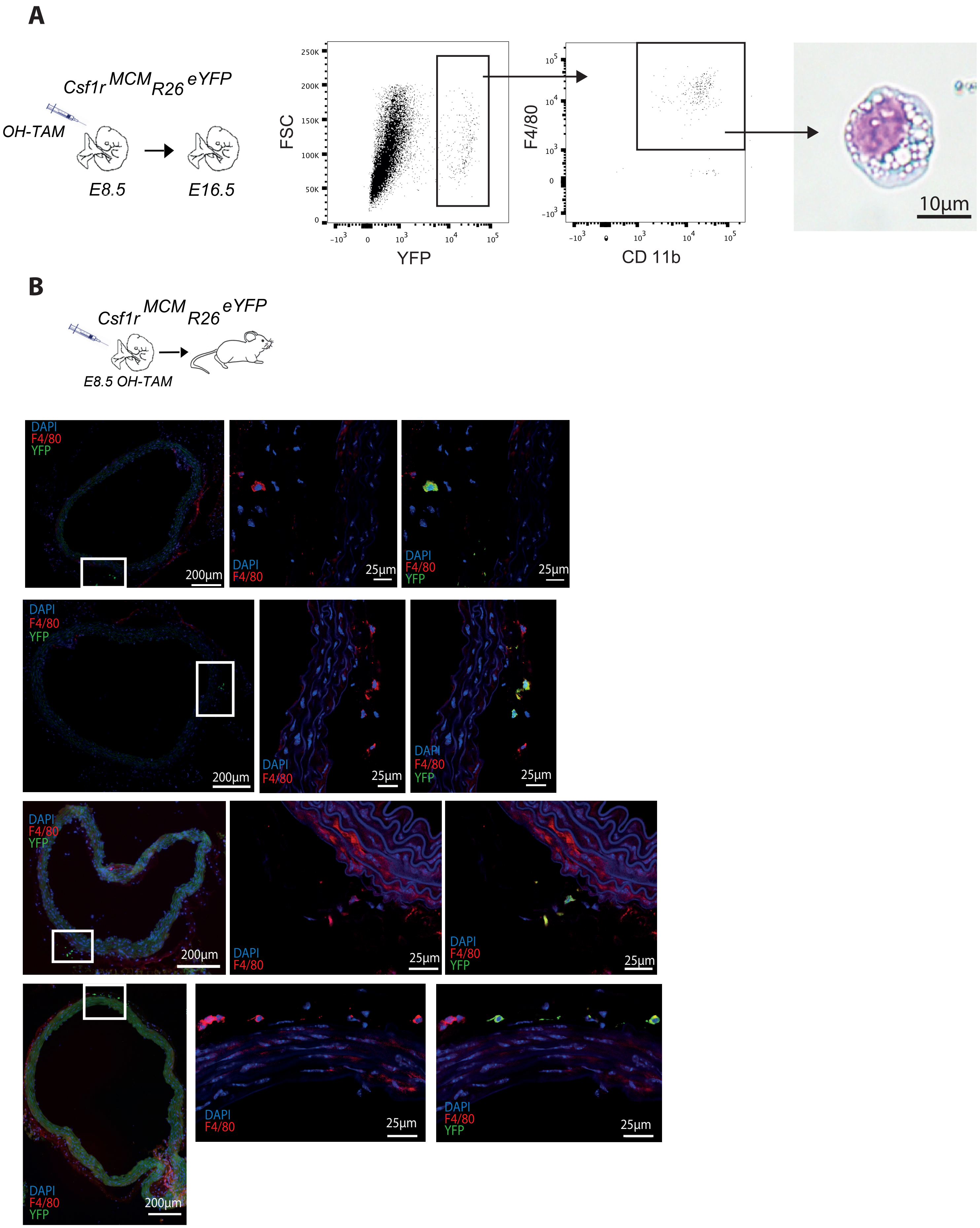

Supplementary Figure 1

Yolk-sac derived macrophages persist in the aorta into adulthood

(A) Examplary FACS analysis of the aorta harvested from a E16.5 embryo after i.p. injection of hydroxytamoxifen at E8.5 into a pregnant *Csf1r*^{MerCreMer}*Rosa26*^{eYFP} mouse confirming the typical macrophage morphology after sorting, cytospin and May-Grünwald-Giemsa staining (n=2; 2 individual experiments); (B) Exemplary immunohistological stainings from 12 month-old offspring who had received an i.p. injection of hydroxytamoxifen at E8.5 during pregnancy. Labelled macrophages were exclusively found in the adventitia. Scale bar as depicted in images.

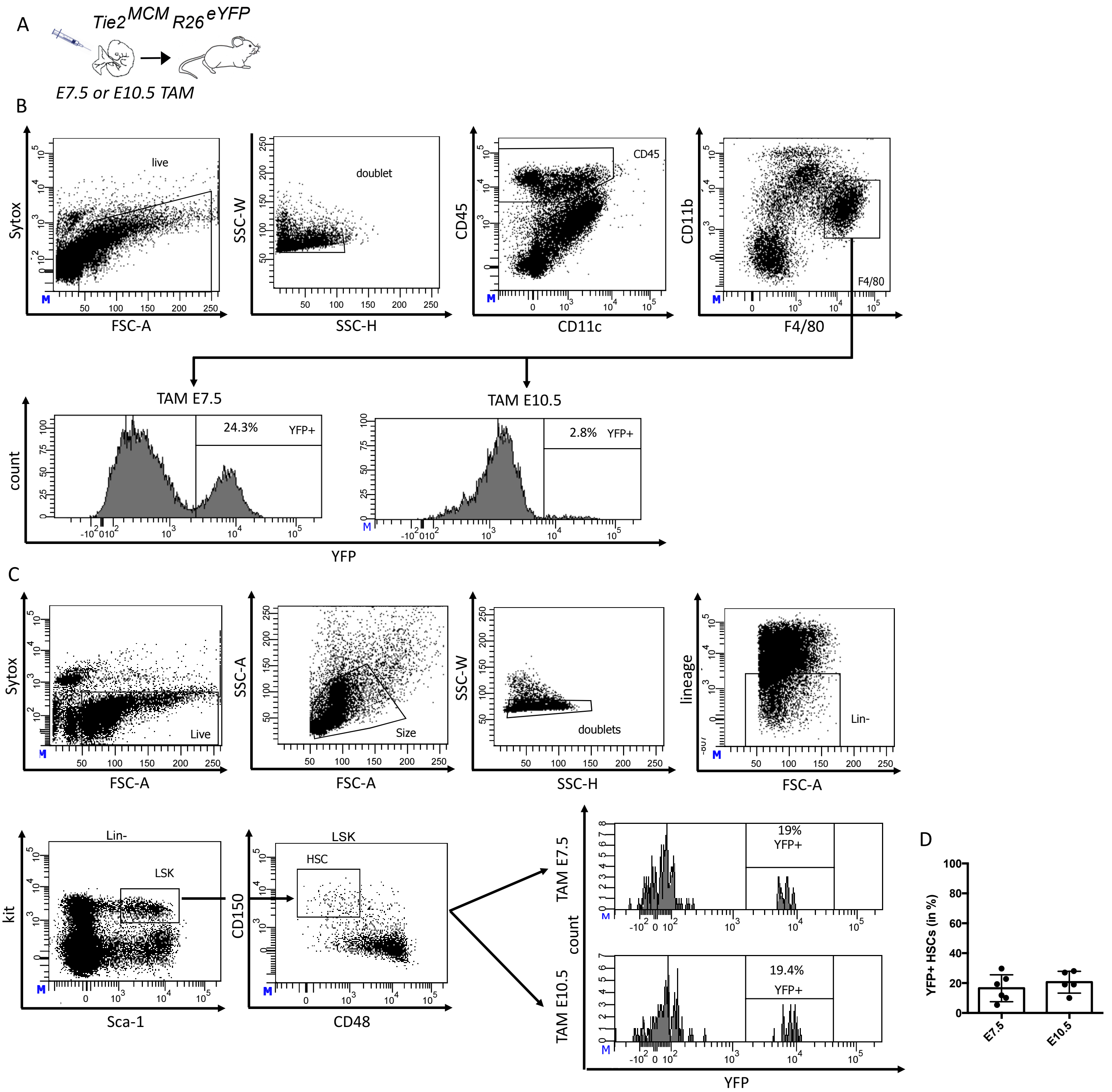

Supplementary Figure 2

Pulse-labelling of liver macrophages and HSCs in *Tie2^{MCM}Rosa26^{eYFP}* mice

(A) Schematic graph of pulse labelling in pregnant *Tie2^{MCM}Rosa26^{eYFP}* mice by oral application of tamoxifen (E7.5 or E10.5), (B) gating strategy to identify liver macrophages (live/singlets/CD45+/CD11c-/CD11b^{lo}/F4/80^{hi}) and their expression of YFP, (C) gating strategy of HSCs (live/singlets/lin(CD11b/CD19/CD3e/CD4/CD8a/Gr-1/Ter119)-/kit+/Sca-1+/CD150+/CD48-) and their expression of YFP, (D) percentage of YFP+ HSCs in 3 month old mice injected at E7.5 and E10.5 (n=6 for E7.5 and n=5 for E10.5; 2 independent experiments at each time-point). Mean ± SD is shown.

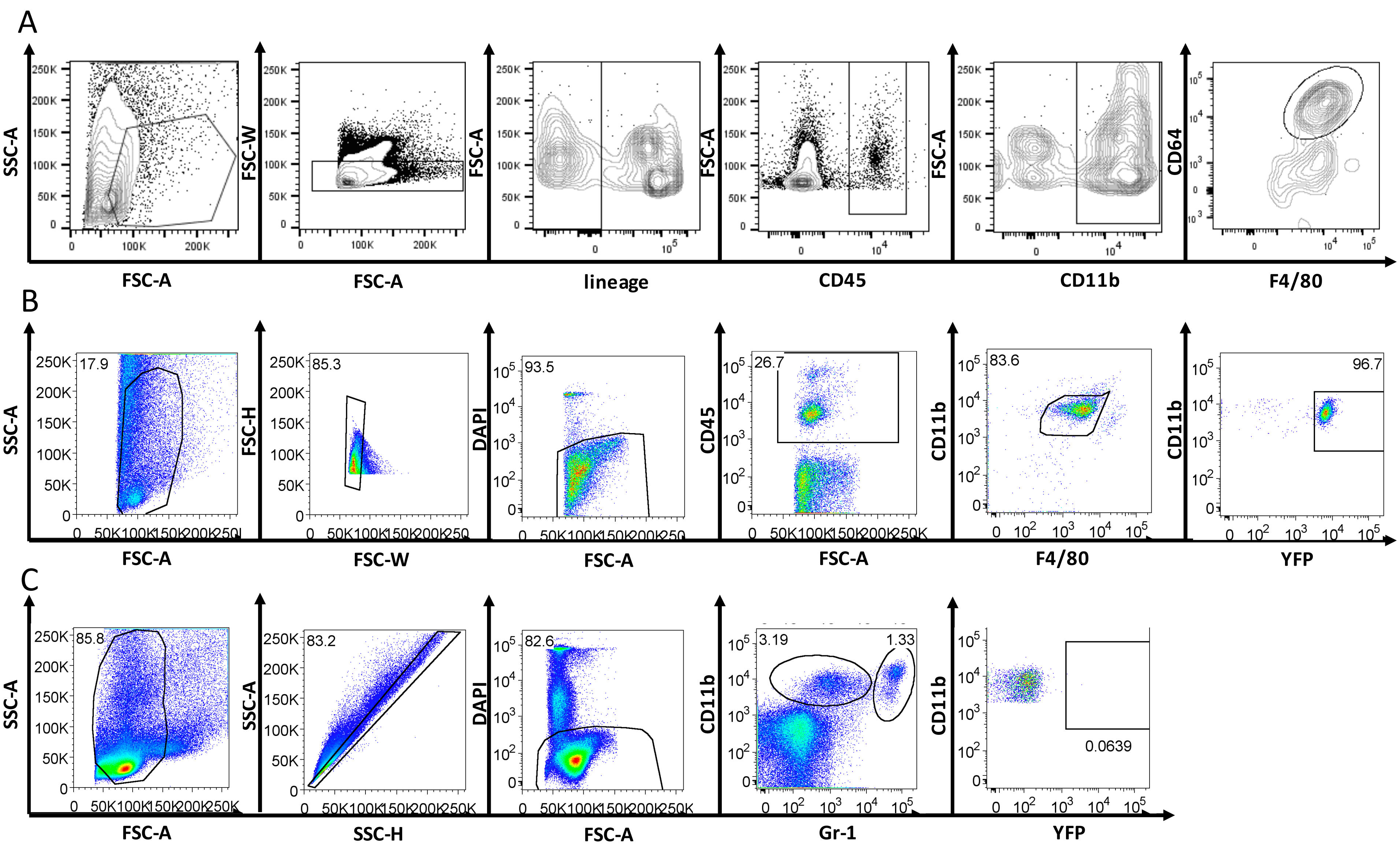

Supplementary Figure 3

Gating strategy of adventitial macrophages, microglia and blood monocytes

(A) Gating strategy of adventital macrophages (single CD45+, lin-(CD11c, SiglecF, Ter119, Ly6g), CD11b+, F4/80+ cells) (B) gating strategy of microglia (live/singlets/ CD45+/CD11b+/F4/80+) and (C) gating strategy of blood monocytes (live/singlets/CD11b^{hi}, Gr-1^{lo})

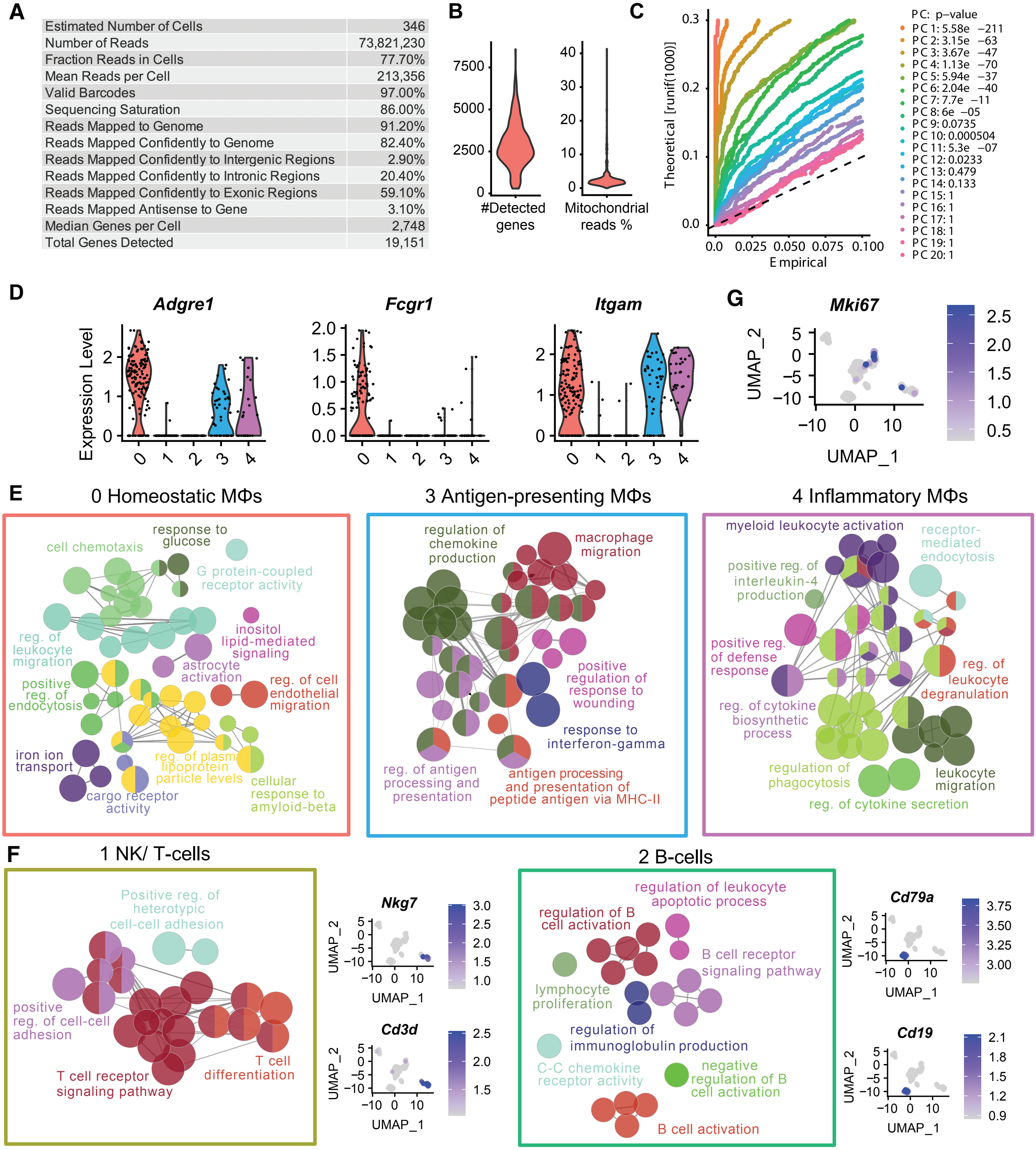

Supplementary Figure 4

Single cell analysis of CD45+ immune cells in *Rank^{Cre}Rosa26^{eYFP}* mice in steady state

(A) Sequencing data quality metrics of steady state adventitial sample. (B) Violin plots depicting distribution of detected genes and percentage of mitochondrial read content used for preprocessing in Seurat. (C) JackStraw analysis of principle components. PCs 1 to 12 were used for downstream analysis. (D) Violin plots showing the expression of general macrophage markers *Adgre1* (F4/80), *Fcgr1* (Cd64) and *Itgam* (CD11b). (E) Expression of *Mki67* as a marker of proliferative cells. Proliferation is mainly limited to cluster 3. (F-G) GO Biological processes (P-value<0.05, GO tree levels 3 to 8) associated with different macrophage populations (F) and other leukocyte cluster (G) and feature plots depicting representative marker genes of each cluster. ClueGo performs the enrichment test based on the hypergeometric distribution and Bonferroni correction

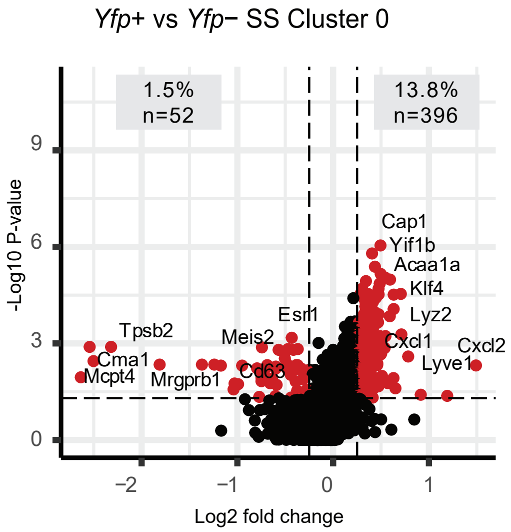

Supplementary Figure 5

Differential gene expression of *Yfp*+ and *Yfp-* macrophages in Cluster 0 at steady state

Volcano plot shows statistical significance (P value) versus magnitude of change (fold change) of genes expressed by *Yfp*+ and *Yfp-* macrophages in cluster 0. Differentially expressed genes (p<0.05) based on Wilcoxon Rank Sum test are indicated in red and their percentage and absolute number is given.

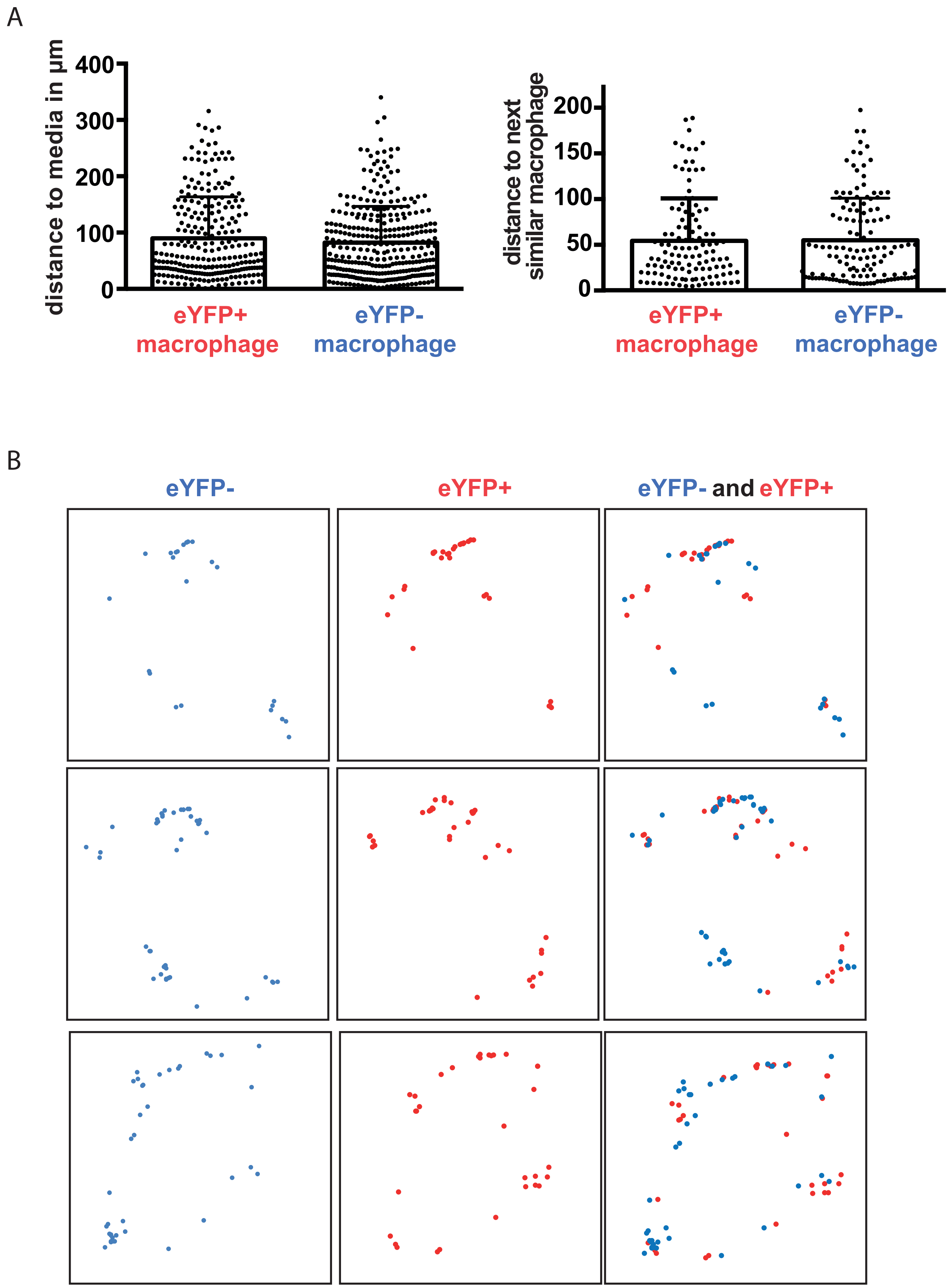

Supplementary Figure 6

Stochastic distribution of adventitial eYFP+ and eYFP- macrophages in *Flt3^{Cre}Rosa26^{eYFP}* mice

(A) Analysis of the macrophage-media distance (n = 330 for eYFP- macrophages, n = 251 for eYFP+ macrophages) as well as the distance to the next macrophage of equal YFP expression (n = 160 for eYFP- macrophages, n = 137 for eYFP+ macropahges) in aortas of *Flt3^{Cre}Rosa26^{eYFP}* mice (n = 3, 3 individual experiments) and (B) their distribution throughout the aorta.

A

N° of analysed macrophages	Percentage of YFP+ macrophages
4.380	45,2%
2.811	44,4%
2.221	51,8%
1.653	52,4%

B

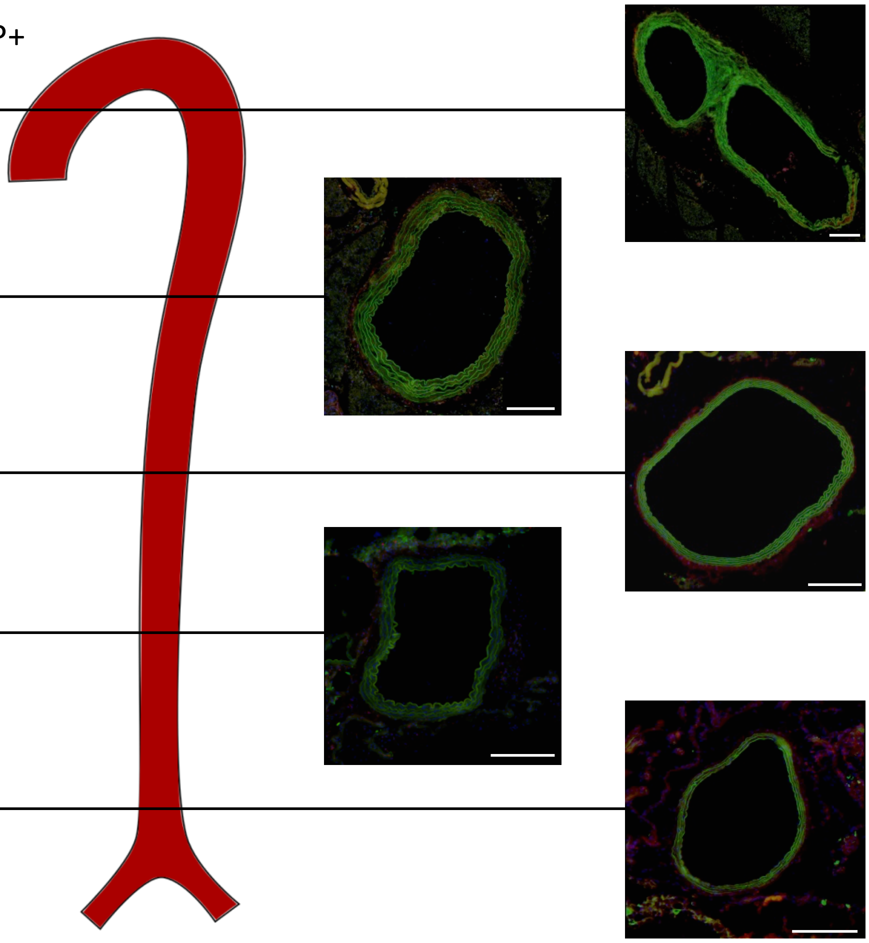

Supplementary Figure 7

Equal distribution of macrophages in different anatomic regions of the aorta in *Flt3^{Cre}Rosa26^{eYFP}* mice

(A) Analysis of 11.065 adventitial macrophages (n=14) in different regions the aorta of *Flt3^{Cre}Rosa26^{eYFP}* mice and percentage of YFP-expression in the defined regions. Scale bar represents 200µm.

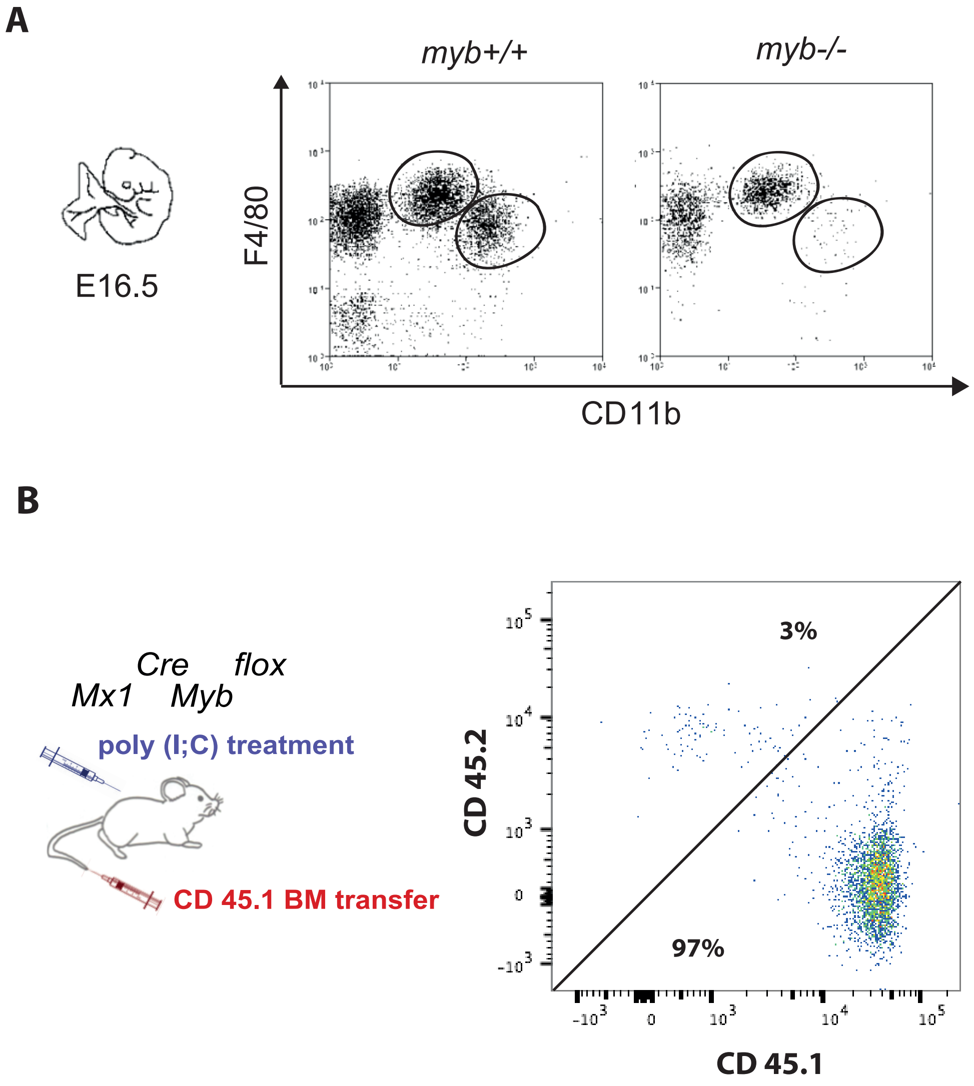

Supplementary Figure 8

Genetic BM-ablation model to generate CD45.1/.2 BM-chimeras

(A) Flow cytometry of aortas harvested from *Myb*$^{+/+}$ and *Myb*$^{-/-}$ mice on E16.5 showing a *myb*-independent F4/80^{hi} macrophage population. (B) exemplary flow cytometry showing the expression of CD45.1 and CD45.2 in CD115^{+} blood monocytes 1 month after BM-transplantation of CD45.1 BM into CD45.2 *Mx1*^{Cre}*Myb*$^{fl/fl}$ mice.

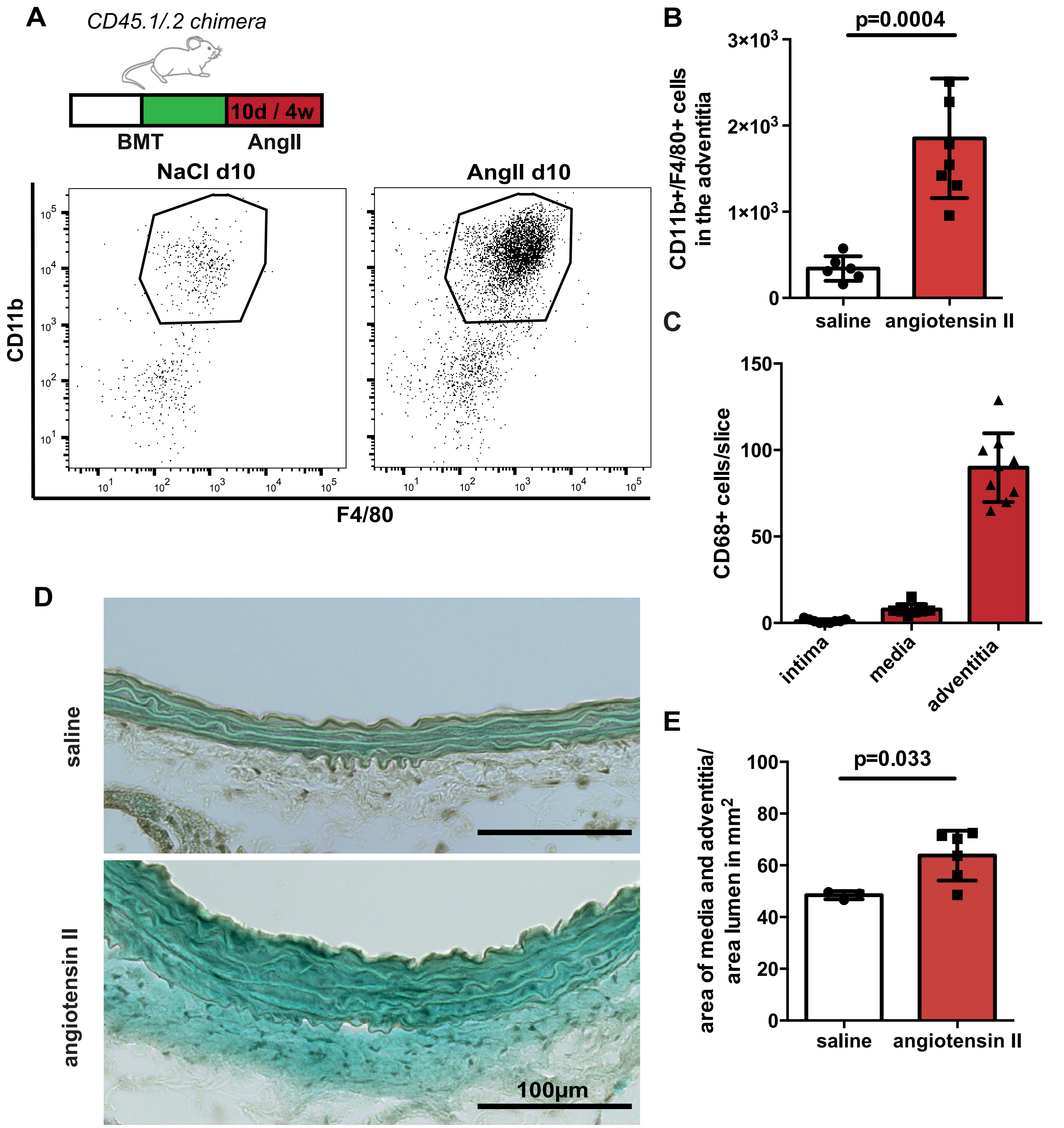

Supplementary Figure 9

Angiotensin II treatment induces arterial inflammation and vascular fibrosis

(A) Representative flow cytometry of single $CD45^+/lin^-/CD11b^+/F4/80^+$ macrophages in the adventitia after 10 days of angiotensin II infusion and (B) quantification of macrophages in saline as well as angiotensin II treated mice (n=6 for saline and n=7 for AngII; each 3 independent experiments). (C) Histological quantification of macrophages (CD68+ cells) in different anatomical regions (n=3, 3 slices per animal are shown, 3 independent experiments). (D) Exemplary masson-trichrome staining of aortas of mice treated with saline or angiotensin II (28 days) and (E) quantification of the fibrotic tissue in the adventitia and media in masson-trichrome stainings (n=3 for saline and n=6 for AngII; each 2 indipendent experiments). Two-sided tests were performed. Mean ± SD is shown.

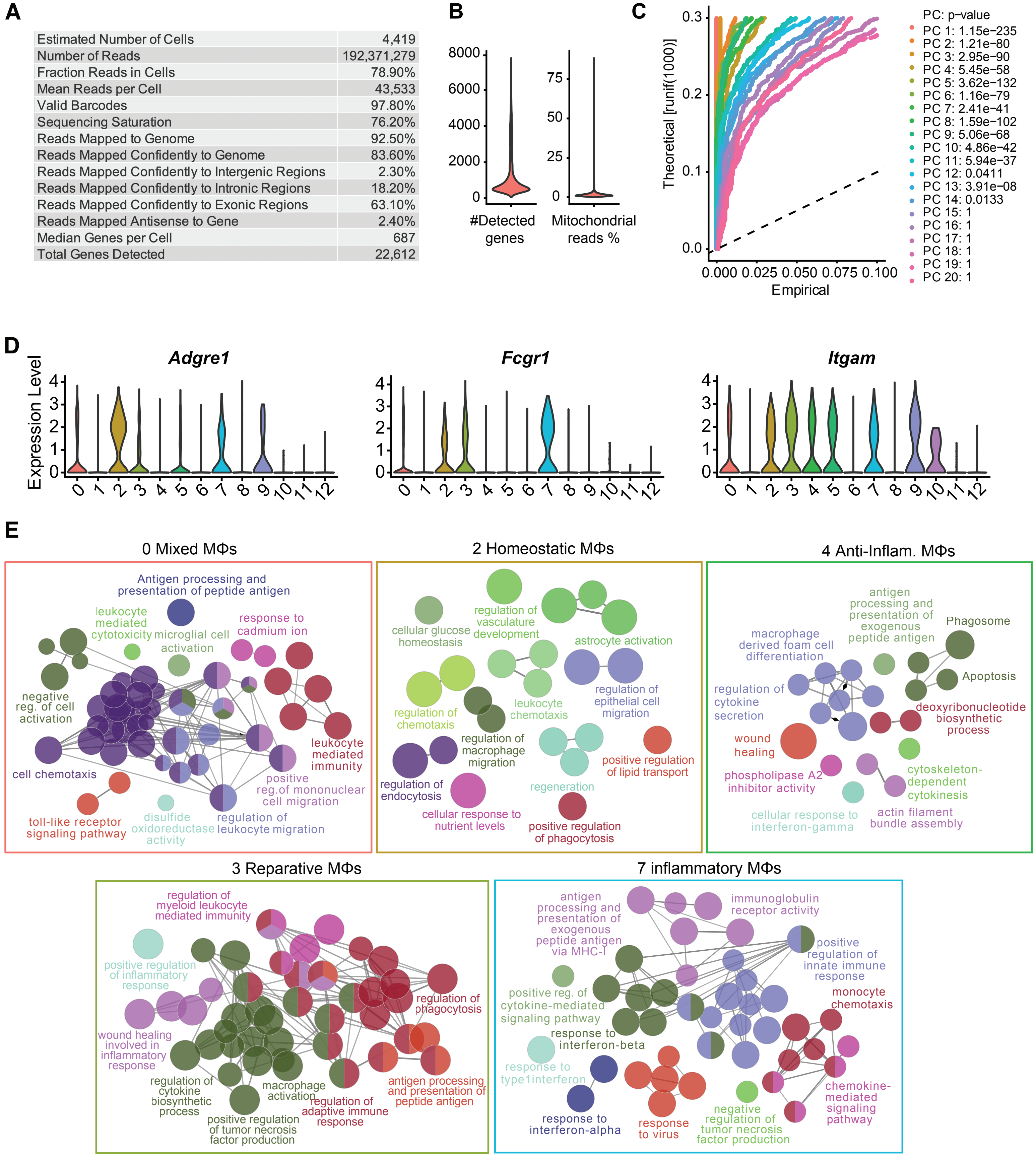

Supplementary Figure 10

Single cell analysis of CD45+ immune cells in *Rank^{Cre}Rosa26^{eYFP}* mice in AngII-induced inflammation

(A) Sequencing data quality metrics of CD45+ cells from adventitias of angiotensin II treated mice. (B) Violin plots depicting distribution of detected genes and percentage of mitochondrial read content used for preprocessing in Seurat. (C) JackStraw analysis of principle components. PCs 1 to 14 were used for downstream analysis. (D) Violin plots showing the expression of general macrophage markers *Adgre1* (F4/80), *Fcgr1* (Cd64) and *Itgam* (CD11b) through different leukocyte clusters. (E) GO Biological processes (P-value<0.05, GO tree levels 3 to 8) associated with different macrophage populations calculated using ClueGO. The markers in all panels were identified using the Wilcoxon Rank Sum test. ClueGo performs the enrichment test based on the hypergeometric distribution and Bonferroni correction.

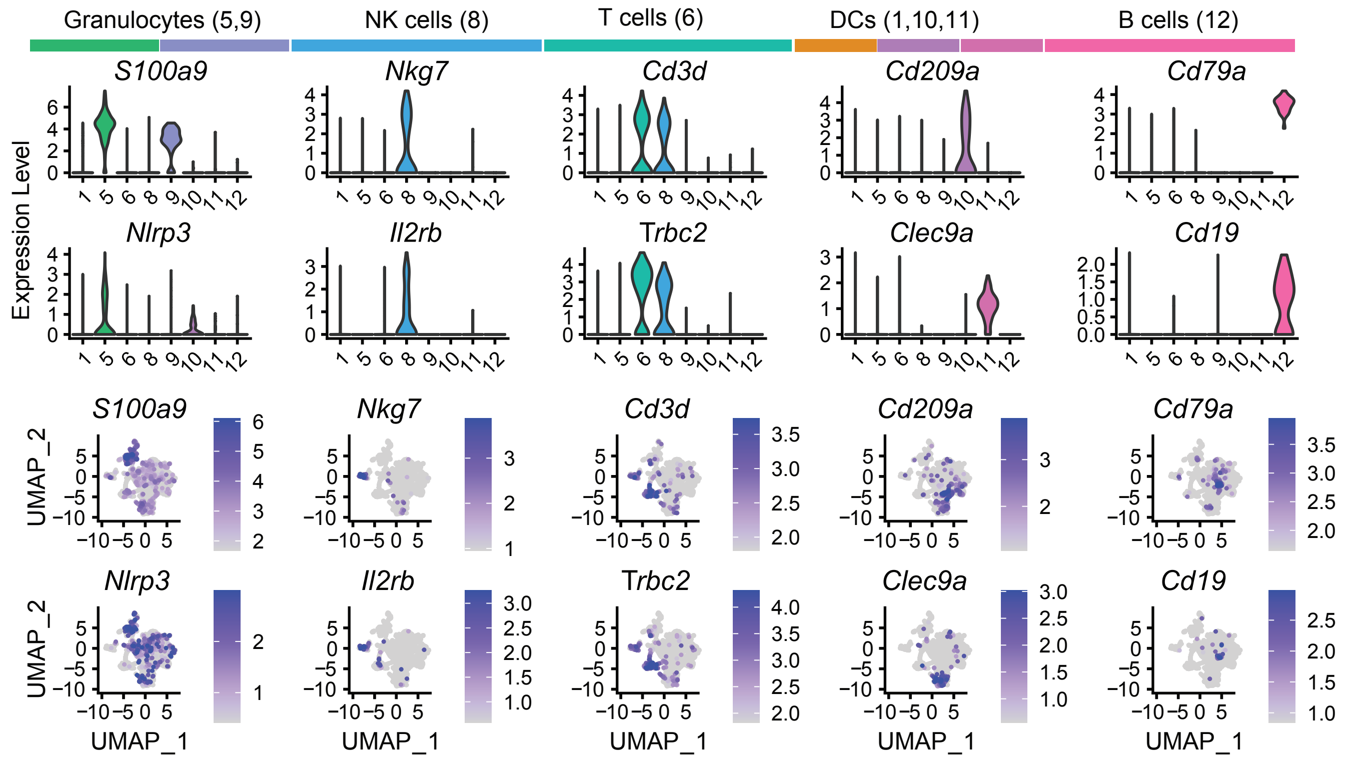

Supplementary Figure 11

Single cell analysis of CD45+ immune cells in *Rank^Cre^Rosa26^eYFP^* mice in AngII-induced inflammation

Violin and feature plots depicting specific marker genes of differnt leukocytes in the adventitia under AngII stimulation

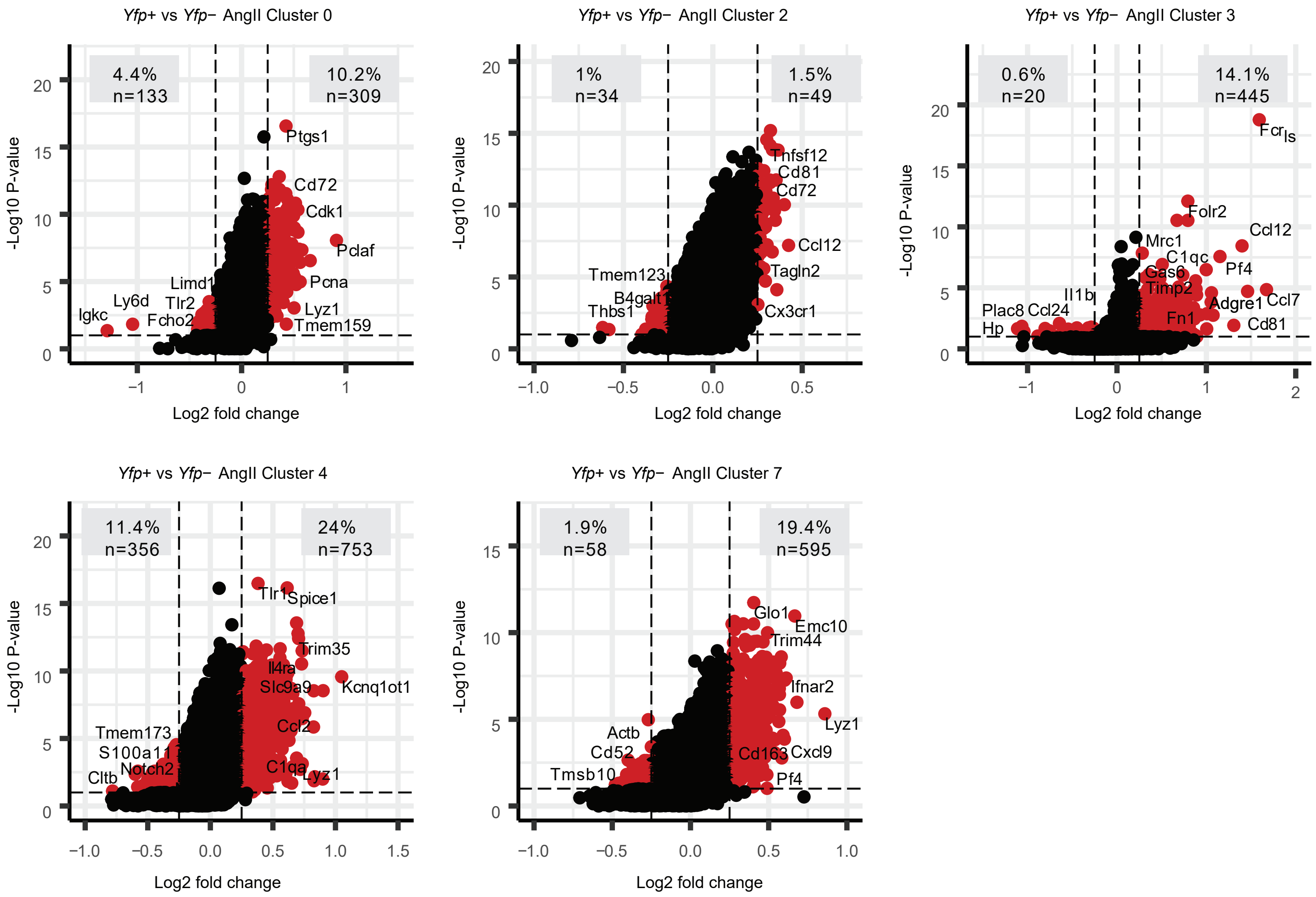

Supplementary Figure 12

Differential gene expression of *Yfp*+ and *Yfp-* macrophages in AngII inflammation

Volcano plots show statistical significance (P value) versus magnitude of change (fold change) of genes expressed by *Yfp*+ and *Yfp-* macrophages in clusters 0,2,3,4,7. Differentially expressed genes ($p<0.05$), identified using Wilcoxon Rank Sum test, are indicated in red and their percentage and absolute number is given.

Cluster-overlapping genes of *Yfp*+ macrophages

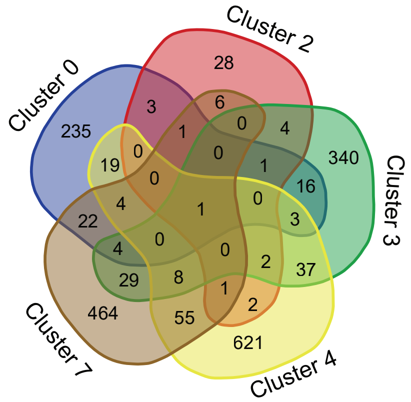

Common between clusters:	Number of common genes	Genes
All macrophages clusters	1	Ccl12
Cluster 0 Cluster 2 Cluster 3	1	Cd81
Cluster 0 Cluster 2 Cluster 7	1	Cx3cr1
Cluster 0 Cluster 3 Cluster 4	3	C1qa C1qc Cmc1
Cluster 0 Cluster 3 Cluster 7	4	Blvrb Fcrls Xpo1 Matr3
Cluster 0 Cluster 4 Cluster 7	4	Abhd12 Rfc1 Lyz1 Etfb
Cluster 2 Cluster 3 Cluster 4	2	Ncl Ltc4s
Cluster 2 Cluster 4 Cluster 7	1	Lair1
Cluster 3 Cluster 4 Cluster 7	8	Maf Cdk6 Pf4 Cemip2 Tmem165 Derl2 Ccl2 Timm10
Cluster 0 Cluster 2	3	Usp1 Mphosph8 Cd72
Cluster 0 Cluster 3	16	Ddx39 Lacc1 Nudc Leng1 Ntpcr Brd3 Lsm7 Impdh1 Gcnt1 Tax1bp3 C1qb Vkorc1 Ntan1 Ccl4 Sarnp Anp32a
Cluster 0 Cluster 4	19	Sorl1 Capns1 Senp6 Aif1 Atp5o.1 Stk38 Txndc15 Shtn1 Plk3 Wdr83os Polr1c Ptgs1 Efr3a Hacd2 Taz Efcab14 Mtif2 Acp5 Naip2
Cluster 0 Cluster 7	22	Nt5c2 Pcbp2 Srsf6 Epn1 Ccl3 Eif4g2 Smc6 Mitf Acad8 Rab7 Frg1 Jak1 E2f4 Ndufa8 Chm Smc1a Mea1 Ube2s Sphk2 Bud23 Lamp1 Scimp
Cluster 2 Cluster 3	4	Cct4 Ldha Slamf9 Casd1
Cluster 2 Cluster 4	2	Rwdd1 Bag1
Cluster 2 Cluster 7	6	Dnajc13 Myo5a R3hdm4 P2ry6 Snrpd1 Jun
Cluster 3 Cluster 4	37	Mtx1 Akt1 Ccl7 Degs1 Rxrb Tspan32 Eif2b4 C3ar1 Igfbp4 Msh3 2310009A05Rik Hopx 9530068E07Rik Mettl1 Mfap1a Sel1l Folr2 Swi5 Ago1 Ptpmt1 Adrb2 Vps29 Bcl2l13 Cenpb Tiparp Timp2 Gm8995 Surf4 Dnajc25 Rundc1 Kdm6a Tnfsf9 Srrt Adgre1 Sart3 Sept9 Rapgef1
Cluster 3 Cluster 7	29	Stard8 Itga6 Fam3a Mvb12b mt-Nd1 Smchd1 Elmo1 Smim10l1 Cetn2 Nxpe5 Kctd12 Wdr43 Cct3 Msmo1 mt-Nd2 Thyn1 Emc10 Trim8 Supt6 mt-Cytb Stab1 Pdk1 Gtf2i Dctn4 Mid1ip1 Vma21 Srf Safb2 Nfkbiz
Cluster 4 Cluster 7	55	Ep400 Supt4a Sdad1 Plscr3 Cpsf3 Ddx21 Rbm22 Atp2b1 Gpbp1 Dpm1 Parp8 Ms4a7 Pnn Commd8 Sertad3 Adam19 Pou2f2 Wsb1 Cnp Pik3ca Dnm2 Ube2h Kdm5a Rnf13 Rab28 Zadh2 Golgb1 Prkx Sec24a Rcor3 Gpr65 Parp14 Hs6st1 Vrk2 Hebp1 G3bp2 Trip12 Ssbp4 Pcf11 Ndufab1 Uqcrh Faim Acot2 Kctd10 Gapvd1 Ccr1 Zfp871 Psmd4 Milr1 Smpdl3b Fnbp1l Tgfbr2 Gsap Madd Rin2

Cluster-overlapping genes of *Yfp-* macrophages

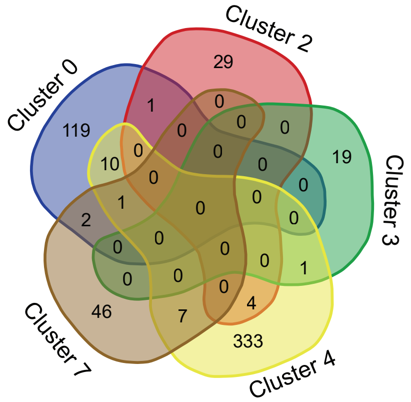

Common between clusters:	Number of common genes	Genes
Cluster 0 Cluster 4 Cluster 7	1	Map2k3
Cluster 0 Cluster 2	1	Tmem123
Cluster 0 Cluster 4	10	Fam107b Paip2 Rbbp6 Ankrd17 Ash1l Reep3 Phf20l1 Osbpl9 Crip1 Stat3
Cluster 0 Cluster 7	2	BC031181 Cebpg
Cluster 2 Cluster 4	4	Zfp207 Rel Exoc5 Tomm5
Cluster 3 Cluster 4	1	Fosl2
Cluster 4 Cluster 7	7	Dpm2 Tmsb10 Rad23a AI413582 Babam1 Bola3 Rp2

Supplementary Figure 13

Overlap of differentially expressed genes in *Yfp*+ and *Yfp-* macrophages across clusters in AngII inflammation

Venn diagrams (left) indicate relation of clusters and the number of common genes between clusters. Tables (right) indicate gene names in overlapping clusters.

Supplementary Table 1: Genotyping

1 FltCre (generic Cre-protocol)

1	AllCreF	CGA TGC AAC GAG TGA TGA GG
2	AllCreR	CGC ATA ACC AGT GAA ACA GC

2 cMyb

1	Myb_WT5’	CCA TGC GTC GCA AGG TGG AAC
2	Myb_WT3’	GTG CTT CGG CGA TGT GGT AA
3	Myb_MUT3’	TGG CCG CTT TTC TGG ATT CAT C

3 cMybflox

1	LoxP_1	ATC TGA AGA AAA TGA ATT GA
2	LoxP_2	GCA TCA GCT CGA TGA TAA GCA

4 Mx1-Cre

1	30250	GTG AGT TTC GTT TCT GAG CTC C
2	14314	CGG TTA TTC AAC TTG CAC CA

5 Csf1r-MerCreMer

1	CFMS Promoter	TCA TTC CAG AAC CAG AGC
2	MER Reverse	GAT CGT GTT GGG GAA GCC

6 Tie2-MerCreMer

1	WT5’	CAT CGC ATA CCA TAC ATA GGT GGA GG
2	MUT3’	AAT CAA GGG TCC CCA AAC TCA C
3	WT3’	GAG GCA GCA TCT GTC TAC AAG AGATGG

7 c-Kit-MerCreMer (generic Cre)

1	Cre-F	GGC GTT TTC TGA GCA TAC CT
2	Cre-R	CTA CAC CAG AGA CGG AAA TCC

8 Rank-Cre (Waskow)

1	Rank-Cre Waskow fw	AAC CTG AGG ATG TGA GGG ACT A
2	Rank-Cre Waskow rev	GTC AAA GTC AGT GCG TTC AAA G

9 Rosa26-mT/mG

1	oIMR7318	CTC TGC TGC CTC CTG GCT TCT
2	oIMR7319	CGA GGC GGA TCA CAA GCA ATA
3	oIMR7320	TCA ATG GGC GGG GGT CGT T

10 Rosa26-eYFP

1	oIMR4982	AAG ACC GCG AAG AGT TTG TC
2	oIMR8545	AAA GTC GCT CTG AGT TGT TATC
3	oIMR8546	GGA GCG GGA GAA ATG GAT ATG

11 Rosa26-eGFP

1	PGKpA-F	GAT CAG CAG CCT CTG TTC CACA
2	EGFP-5R	CGC TGA ACT TGT GGC CGT TTA C

Supplementary Table 2: Antibody List

Antibody	Dilution	Clone	Fluorescence	Company	Catalog number
**Flow Cytometry**					
**F4/80**	1:50	BM8	APC	eBioscience	17-4801-82
**CD 48**	1:100	HM 48-1	PE	eBioscience	12-0481-82
**CD115**	1:100	AFS98	BV421	Biolegend	135513
**CD117**	1:100	ACK-2	AF700	eBioscience	56117282
**CD11b**	1:100	M1/70	PE-Cy7	BD Biosciences	552850
**CD11b**	1:100	M1/70.15	bio	Invitrogen	13-0112-82
**CD11b**	1:100	M1/70	PeCy7	eBio	25-0112-82
**CD11c**	1:100	N418	PE	Biolegend	561044
**CD11c**	1:100	N418	PerCP-Cy5.5	eBio	45-0114-82
**CD150**	1:100	TC15-12F12.2	BV605	BioLegend	115903
**CD16/CD32 (FcγRIII/FcγRII)**	1:50	2.4G2	uncoupled	BD Biosciences	553142
**CD64**	1:100	X54-5/7.1	APC	Biolegend	139306
**CD3e**	1:100	145-2C11	bio	eBio	13-0031-82
**CD4**	1:100	GK1.5	bio	Ph	553728
**CD45**	1:100	cl.30-F11	eF780 = APCCy7	eBioscience	47-0451-82
**CD45.1**	1:100	A20	Fitc	BD Biosciences	110706
**CD45.2**	1:100	104	APC-Cy7	BD Biosciences	560694
**CD68**	1:200	FA11	uncoupled	BioRad	MCA1957
**CD8a**	1:100	53-6.7	bio	eBio	13-0081-82
**F4/80**	1:100	BM8	BV421	Biolegend	123131
**Gr-1**	1:100	RB6-8C5	bio	eBio	13-5931-82
**Ly6G**	1:100	1A8	PE	Biolegend	127608
**NK1.1**	1:100	PK136	PE	Biolegend	108708
**SA**	1:100		APC Cy7	Ph	554063
**Sca-1**	1:100	D7	PerCP-Cy5.5	eBioscience	45-5981-82
**Siglec-F**	1:100	E50-2440	PE	BD Biosciences	552126
**TCR-ß**	1:100	1B3.3	PE	Biolegend	109208
**Ter119**	1:100	Ter119	PE	Biolegend	116208
**Ter119**	1:100	Ter119	PE	eBio	12592182
**Immunohistology**					
**anti-GFP**	1:100	Polyclonal, Rabbit	uncoupled	Invitrogen	A-11122
**anti-GFP**	1:100	Polyclonal, Goat	uncoupled	abcam	Ab6673
**CD45.2**	1:50	104	uncoupled	Merck Millipore	MABF441
**F4/80**	1:50	BM8	uncoupled	Invitrogen	14-4801-85
**CD68**	1:200	FA11	uncoupled	BioRad	MCA1957
**Goat IgG**	1:100	Polyclonal, Goat	uncoupled	Santa Cruz	Sc-2028
**Isotype IgG2a, κ**	1:50	eBR2a	uncoupled	Invitrogen	14-4321-82
**Isotype IgG2a, κ**	1:50	MOPC-173	uncoupled	BioLegend	400201
**Rabbit Immunoglobulin Fraction**	1:100	Polyclonal, Rabbit	uncoupled	Dako	X0936
**AF350 goat anti-rabbit**	1:200	Polyclonal	Alexa Fluor 350	Invitrogen	A-11046
**AF488 donkey anti-goat**	1:200	Polyclonal	Alexa Fluor 488	Invitrogen	A-11055
**AF488 goat anti-mouse**	1:200	Polyclonal	Alexa Fluor 488	Invitrogen	A-11001

**AF488 goat anti-rabbit**	1:200	Polyclonal	Alexa Fluor 488	Invitrogen	A-11034
**AF594 goat anti-rabbit**	1:200	Polyclonal	Alexa Fluor 594	Invitrogen	A-11037
**AF647 goat anti-rabbit**	1:200	Polyclonal	Alexa Fluor 647	Invitrogen	A-21244
**Cy3 goat anti-rat**	1:200	Polyclonal	Cy3	Jackson Immunoresearch	112-165-003
**Cy5 goat anti-rabbit**	1:200	Polyclonal	Cy5	Jackson Immunoresearch	111-175-144
**Cy5 goat anti-rat**	1:150	Polyclonal	Cy5	Jackson Immunoresearch	112-175-143
**Click-iT™ EdU proliferation kit**	According to manufacturers protocol		Alexa Fluor 594	Thermofisher	C10339
