# Supplementary material for: Ontogeny of arterial macrophages defines their functions in homeostasis and inflammation
Source: Nat Commun. 2020 Sep 11;11:4549. doi: 10.1038/s41467-020-18287-x (PMC7486394; doi:10.1038/s41467-020-18287-x)
Supplement: Supplementary file 3 — Reporting Summary [file 41467_2020_18287_MOESM3_ESM.pdf]

nature research

Corresponding author(s): Christian Schulz

Last updated by author(s): Aug 10, 2020

Reporting Summary

Nature Research wishes to improve the reproducibility of the work that we publish. This form provides structure for consistency and transparency in reporting. For further information on Nature Research policies, see our Editorial Policies and the Editorial Policy Checklist.

Statistics

For all statistical analyses, confirm that the following items are present in the figure legend, table legend, main text, or Methods section.

n/a	Confirmed	
☐	☒	The exact sample size (*n*) for each experimental group/condition, given as a discrete number and unit of measurement
☐	☒	A statement on whether measurements were taken from distinct samples or whether the same sample was measured repeatedly
☐	☒	The statistical test(s) used AND whether they are one- or two-sided<br>*Only common tests should be described solely by name; describe more complex techniques in the Methods section.*
☒	☐	A description of all covariates tested
☒	☐	A description of any assumptions or corrections, such as tests of normality and adjustment for multiple comparisons
☐	☒	A full description of the statistical parameters including central tendency (e.g. means) or other basic estimates (e.g. regression coefficient) AND variation (e.g. standard deviation) or associated estimates of uncertainty (e.g. confidence intervals)
☐	☒	For null hypothesis testing, the test statistic (e.g. *F*, *t*, *r*) with confidence intervals, effect sizes, degrees of freedom and *P* value noted<br>*Give P values as exact values whenever suitable.*
☒	☐	For Bayesian analysis, information on the choice of priors and Markov chain Monte Carlo settings
☒	☐	For hierarchical and complex designs, identification of the appropriate level for tests and full reporting of outcomes
☒	☐	Estimates of effect sizes (e.g. Cohen's *d*, Pearson's *r*), indicating how they were calculated

Our web collection on statistics for biologists contains articles on many of the points above.

Software and code

Policy information about availability of computer code

Data collection	BD Software (different versions, latest version: BD FACSDiva Software v8.0.1) was used to collect flow cytometry data. Sequencing of scRNA was perfomed on a HiSeq1500 sequencer (Illumina, San Diego, USA).
Data analysis	FlowJo Version 10 was used for flow cytometry analysis. GraphPad Prism Version 8.0 was used to generate bar graphs and perform statistical analysis. Analysis of scRNAseq-data was performed using CellRanger (v3.1.0, 10x Genomics) and the R package Seurat (v3.1). Biological pathways from scRNAseq analysis were analyzed using ClueGO (v2.5.6), CluePedia (v1.5.6) and Cytoscape (v3.8.0). Volcano blots were genererated using the R package EnhancedVolcano. Venn diagrams were drawn from Significant genes (P-val<0.05) (using the web tool at http://bioinformatics.psb.ugent.be/webtools/Venn/).

For manuscripts utilizing custom algorithms or software that are central to the research but not yet described in published literature, software must be made available to editors and reviewers. We strongly encourage code deposition in a community repository (e.g. GitHub). See the Nature Research guidelines for submitting code & software for further information.

Data

Policy information about availability of data

All manuscripts must include a data availability statement. This statement should provide the following information, where applicable:

- Accession codes, unique identifiers, or web links for publicly available datasets
- A list of figures that have associated raw data
- A description of any restrictions on data availability

The authors declare that all data supporting the findings in this study are available within the article and its Supplementary Information files. The scRNAseq raw data have been deposited in NCBI GEO under the accession codes GSE155569 [https://www.ncbi.nlm.nih.gov/geo/query/acc.cgi?acc=GSE155569], GSM4706334 [https://www.ncbi.nlm.nih.gov/geo/query/acc.cgi?acc= GSM4706334] and GSM4706335 [https://www.ncbi.nlm.nih.gov/geo/query/acc.cgi?acc= GSM4706335].

Source data for figures 1 (B, C, F, G), 3 (B, D, F, H, J), 4 (A, D, F, G, I), 5 (A, B, D, F), Suppl. Fig. 2D, Suppl. Fig. 6 and Suppl. Fig. 9 (B, C, E) are provided with the paper. There are no restrictions regarding data availability.

Field-specific reporting

Please select the one below that is the best fit for your research. If you are not sure, read the appropriate sections before making your selection.

- [x] Life sciences
- [ ] Behavioural & social sciences
- [ ] Ecological, evolutionary & environmental sciences

For a reference copy of the document with all sections, see nature.com/documents/nr-reporting-summary-flat.pdf

Life sciences study design

All studies must disclose on these points even when the disclosure is negative.

Sample size	Pilot analyses and previous literature were used to determine sample size (Ensan S, Li A, Besla R, et al. Self-renewing resident arterial macrophages arise from embryonic CX3CR1(+) precursors and circulating monocytes immediately after birth. Nat Immunol. 2016;17 (2):159-168. doi:10.1038/ni.3343.; Moore, J. P. et al. M2 macrophage accumulation in the aortic wall during angiotensin II infusion in mice is associated with fibrosis, elastin loss, and elevated blood pressure. Am J Physiol Heart Circ Physiol 309, H906-917, doi:10.1152/ ajpheart.00821.2014 (2015))
Data exclusions	No data were excluded from the analysis.
Replication	All of our analyses have been successfully been reproduced within our laboratory by different team members. To achieve this, we have regulated 'standard operating procedures' for each protocol. In addition, some analyses were carried out by collaborators in different countries. Each experiment was performed at least in two independent using at least 3 animals.
Randomization	Samples were allocated either 1) into experimental groups of Cre+ mice compared to Cre- mice, or 2) in case Cre+ mice were subjected to specific treatments (e.g. PLX feeding, AngII application) we used simple random sampling. Groups were defined at the beginning of the experiments in a prespecified order of "treatment" (e.g. PLX5622 chow") or "verum" (e.g. ctrl chow) which led to e.g. a randomization into "T,V,T,T;V,T,V,T,V".
Blinding	Analysis of histological specimen as well as flow cytometry was conducted in a blinded fashion. ScRNA-Sequencing was performed in a blinded fashion. Analysis of the RNA-Seq Data could not be blinded as the main focus of the analysis was the comparison of YFP+ and YFP- macrophages.

Reporting for specific materials, systems and methods

We require information from authors about some types of materials, experimental systems and methods used in many studies. Here, indicate whether each material, system or method listed is relevant to your study. If you are not sure if a list item applies to your research, read the appropriate section before selecting a response.

Materials & experimental systems

n/a	Involved in the study	
[ ]	[x]	Antibodies
[x]	[ ]	Eukaryotic cell lines
[x]	[ ]	Palaeontology and archaeology
[ ]	[x]	Animals and other organisms
[x]	[ ]	Human research participants
[x]	[ ]	Clinical data
[x]	[ ]	Dual use research of concern

Methods

n/a	Involved in the study	
[x]	[ ]	ChIP-seq
[ ]	[x]	Flow cytometry
[x]	[ ]	MRI-based neuroimaging

Antibodies

Antibodies used	Details of the antibodies are included within the text of within Supplementary Table 2.
Validation	All antibodies are commercial antibodies and have been previously validated (and published) in other mouse studies. The relevant references are available on-line on the respective websites.

Animals and other organisms

Policy information about studies involving animals; ARRIVE guidelines recommended for reporting animal research

Laboratory animals	Flt3Cre, Myb−/−, Mybfl/fl, Mx1Cre, Csf1rMerCreMer, Tie2MCM, c-KitMerCreMer, RankCre (also known as Tnfrsf11aCre), C57Bl6/ CD45.2, C57Bl6/CD45.1, Rosa26mTmG, Rosa26eYFP and Rosa26eGFP reporter mice were used. All primers used for genotyping are

	described in the Supplementary Table 1.
Wild animals	No wild animals were used.
Field-collected samples	No field-collected samples were used.
Ethics oversight	We have complied with all relevant ethical regulations. Animal studies were approved by the local regulatory agency (Regierung von Oberbayern, Munich, Germany), record numbers 55.2.1.54-2532-93-13, 55.2-1-54-2532-55-15, 55.2.1.54-2532-183-16, ROB-55.2-2532.Vet_02-19-17 and ROB-55.2-2532.Vet_02-19-1.

Note that full information on the approval of the study protocol must also be provided in the manuscript.

Flow Cytometry

Plots

Confirm that:

☒ The axis labels state the marker and fluorochrome used (e.g. CD4-FITC).

☒ The axis scales are clearly visible. Include numbers along axes only for bottom left plot of group (a 'group' is an analysis of identical markers).

☒ All plots are contour plots with outliers or pseudocolor plots.

☒ A numerical value for number of cells or percentage (with statistics) is provided.

Methodology

Sample preparation	Adult mice were euthanized by cervical dislocation under anaesthesia. Blood was collected by cardiac puncture from anaesthetized mice (fentanyl (0.05 mg/kg per kg), midazolam (5.0 mg/kg per kg) and medetomidine (0.5 mg/kg per kg)). Under anaesthesia, mice were perfused by gentle intracardiac injection of 10 ml heparinised PBS. The mouse aorta was harvested using a dissecting microscope. After 15 minutes enzymatic digestion with Collagenase II (Worthington Biochemical; 20mg/ml) and Elastase (Worthington Biochemical; 20mg/ml) at 36°C the adventitia was separated from the media and passed through a 70µm filter.<br>Bone marrow: Cells were flushed from femurs, tibias and coxa using PBS suppemented with 5% heat-inactivated FCS in PBS. Cells were filtered through a 20-µm filter (Falcon).<br>Livers were digested with collagenase IV (600U/ml) and DNase I (2,5g/ml) at 37°C in RPMI for 45 minutes, washed and resuspended in HBSS. NPC were isolated by Percoll gradient (50%/25%) centrifugation at 1800g, 15 minutes. The NPC-containing middle layer was collected and washed.
Instrument	BD Biosciences FACSCanto II or BD FACS LSR Fortessa
Software	We used BD software to collect data, and FlowJo V10 was used for analysis.
Cell population abundance	Before RNA-sequencing we assessed population abundance using flow cytometry. Following MACS-enrichment of CD45+ cells, we carried out cell sorting of CD45+ live cells as described. The fraction of CD45+ live cells was 70% among MACS-enriched CD45+ cells.
Gating strategy	Gating strategy of adventital macrophages: single CD45+, lin-(CD11c, SiglecF, Ter119, Ly6g), CD11b+, F4/80+ cells)<br>Gating strategy of microglia: live/singlets/lin(CD11b/CD19/CD3e/CD4/CD8a/Gr-1/Ter119)-/kit+/Sca-1+/CD150+/CD48<br>Gating strategy of blood monocytes: live/singlets/lin(CD11b/CD19/CD3e/CD4/CD8a/Gr-1/Ter119)-/kit+/Sca-1+/CD150+/CD48<br>Gating strategy for liver macrophages: live/singlets/CD45+/CD11c-/CD11blo/F4/80hi<br>Gating strategy for HSCs: live/singlets/lin(CD11b/CD19/CD3e/CD4/CD8a/Gr-1/Ter119)-/kit+/Sca-1+/CD150+/CD48-<br>Gates and quadrants were determined by isotype controls or FMOs for all experiments.<br>For YFP-expression, the negative cell population was determined by Cre- control animals.

☒ Tick this box to confirm that a figure exemplifying the gating strategy is provided in the Supplementary Information.
