# Supplementary material for: Ontogeny of arterial macrophages defines their functions in homeostasis and inflammation
Source: Nat Commun. 2020 Sep 11;11:4549. doi: 10.1038/s41467-020-18287-x (PMC7486394; doi:10.1038/s41467-020-18287-x)
Supplement: Supplementary file 4 — Description of Additional Supplementary Files [file 41467_2020_18287_MOESM4_ESM.pdf]

Description of Supplementary Files

File Name: Supplementary Data 1

Description:
Sheet 1: Cell type specific markers in Seurat-based clustering of adventital immune cells in steady state
Sheet 2: Cell type specific markers in Seurat-based clustering of adventital immune cells in AngII induced inflammation
Sheet 3: Differentially expressed genes (*eYfp*+ vs. *eYfp*- cells) in steady state
Sheet 4: Differentially expressed genes (*eYfp*+ vs. *eYfp*- cells) in AngII induced inflammation
